# Psychological, Behavioral, and Interpersonal Effects and Clinical Implications for Health Systems of the Coronavirus (COVID-19) Pandemic: A Call for Research

**DOI:** 10.3389/fpsyg.2020.02146

**Published:** 2020-09-24

**Authors:** Gianluca Castelnuovo, Andrea De Giorgio, Gian Mauro Manzoni, Darren C. Treadway, Changiz Mohiyeddini

**Affiliations:** ^1^Istituto Auxologico Italiano IRCCS, Psychology Research Laboratory, Ospedale San Giuseppe, Verbania, Italy; ^2^Department of Psychology, Catholic University of Milan, Milan, Italy; ^3^Faculty of Psychology, eCampus University, Novedrate, Italy; ^4^Daemen College, Amherst, NY, United States; ^5^Oakland University William Beaumont School of Medicine, Rochester, MI, United States

**Keywords:** coronavirus, COVID-19, pandemic, clinical psychology, health psychology, mass reactions, resilience, emergency strategies

## Abstract

The novel coronavirus disease (COVID-19) emerged at the end of 2019 and was classified as a pandemic by the World Health Organization (WHO) on March 11, 2020. Both the COVID-19 emergency and the extraordinary measures to contain it have negatively affected the life of billions of people and have threatened individuals and nations. One of the main goals of clinical and health psychology during this pandemic is to investigate the behavioral, cognitive, emotional, and psychobiological responses to the COVID-19 emergency as well as to the preventive measures that have been imposed by governments to limit the contagion, such as social isolation. Psychological research has the responsibility to deliver sound empirical evidence to inform public health policies and to support and advise governments and policymakers in their introduction of sustainable, feasible, and cost-efficient prevention and intervention guidelines. Hence, the goal of this call for research is to stimulate theoretical discussions and empirical investigations on the bio-psycho-social impacts of COVID-19 for individuals, groups, and nations. We invite contributions that address the challenges that the COVID-19 emergency has imposed on couples, families, and social systems. In addition, we call for studies that assess the specific effects of the COVID-19 pandemic on highly vulnerable populations such as children, adolescents, pregnant women, patients suffering from chronic and life-threatening conditions, healthcare workers, and elderly citizens. Papers focusing on the impact of emotion regulation and coping strategies are encouraged. Original research, data reports, study protocols, single case reports and community case studies, theoretical perspectives, and viewpoints are invited to help improve our understanding of the COVID-19 pandemic.

## Paper

The novel coronavirus disease (COVID-19) emerged at the end of 2019 and was classified as a pandemic by the World Health Organization (WHO) on March 11, 2020. COVID-19 is a respiratory disease with a very high transmissibility and mortality rate. Therefore, governments and health agencies declared public health emergencies. Indeed it is very important to take into account that people are facing an unprecedented situation. For example, in several countries (e.g., Italy, Spain, Germany, and Turkey), this pandemic has led to a lockdown and quarantine of the entire country.

Obviously, both the COVID-19 emergency and the extraordinary measures to contain it have negatively affected the life of billions of people. The COVID-19 pandemic has threatened individuals, nations, and international relationships. Therefore, the WHO has issued guidelines for managing to contain, mitigate, and limit the horrendous negative impacts of this pandemic. However, it is our contention that psychological preventive and therapeutic measures are just as crucial in facing the COVID-19 pandemic.

Thankfully, academic and social institutions around the world have offered online platforms to provide psychological counseling for confirmed patients, patients with suspected infection, quarantined family members, at-risk individuals, healthcare workers, and first responders. Online mental health services could provide cheap and feasible solutions, taking into account the enormous bio-psycho-social costs of the pandemic (Duan and Zhu, 2020; Liu et al., 2020; Xiang et al., 2020).

One of the main goals of clinical psychology and health psychology during and in the aftermath of this pandemic is to investigate the behavioral, cognitive, emotional, and psychobiological responses to the COVID-19 emergency and to the preventive measures that have been imposed by governments to limit the contagion, such as social isolation. In addition, psychological research must deliver sound empirical evidence to inform public health policies and to support and advise governments and policymakers in their introduction of sustainable, feasible, and cost-efficient prevention and intervention guidelines. Hence, the goal of this call for research is to initiate and stimulate theoretical discussions and empirical investigations on the bio-psycho-social impacts of COVID-19 for individuals, groups, and nations. Furthermore, we invite contributions that address the challenges that COVID-19 has imposed on couples, families, and social systems. In addition, we call for studies that assess the specific effects of the COVID-19 pandemic on highly vulnerable populations such as children, adolescents, pregnant women, patients suffering from chronic and life-threatening conditions, healthcare workers, and elderly citizens. Papers addressing the impact of emotion regulation (De Giorgio, 2016) and coping strategies are encouraged. Even when people face the same stressor all over the world, individuals differ enormously at multiple levels, from degrees of exposure to appraisal and coping ways, as well as in their social, family, and work settings, which can moderate as well as mediate the effect of the COVID-19 emergency on people’s health and well-being.

The broad scope of this call for research allows us to invite papers that address the use of new technologies for the implementation of psychological prevention and protocols in healthcare, clinical, social, educational, and work settings, including telepsychology and mHealth-based experiences (Castelnuovo et al., 2015; Smith et al., 2020), taking into account that physicians and psychologists around the globe have been forced to modify their traditional settings to provide online and remote services (Castelnuovo, 2017).

Moving from a deficit-oriented approach toward a positive psychology of trauma and loss, we also encourage papers that address the COVID-19 emergency as a “chance” to foster individual coping skills, enhance social relations, modify healthcare systems, and fight health disparities—for example, reducing preventable differences in the burden of disease, such as considering and supporting elderly patients or other frail populations.

Particular attention has to be dedicated to those patients severely affected by COVID-19 and those who require hospitalization. According to Jiang et al. (2020), the psychological needs of various groups could differ: “the guiding principles divide the population affected by NCP (new coronavirus pneumonia) into 4 levels and require the first-level population to be the focus of PCI (psychological crisis intervention) (Chinese Society of Psychiatry, 2020; Ma et al., 2020):

(1) Patients with severe symptoms of NCP, front-line medical staff, CDC researchers or administrative staff;
(2) Patients with mild symptoms of NCP, close contacts, suspected patients, or patients with fever who come to the hospital for treatment;
(3) People related to the first- and the second-level populations, such as family members, colleagues, or friends and rescuers, such as commanders, administrative staff, or volunteers;
(4) People in affected areas, susceptible groups, or the general public” (pp. 2 and 3).

In summary, original research, data reports, study protocols, single case reports and community case studies, theoretical perspectives, and viewpoints are invited to help improve our understanding of the psychological, social, and behavioral correlates of the COVID-19 pandemic.

The important subject areas of this research topic include:

• Individual, family, and interpersonal coping with the COVID-19 emergency,
• Risk factors for psychological distress at the individual, family, interpersonal, and cultural level (e.g., activity restriction and reduction of pleasant events, personality traits, hypochondria and cyberchondria, mental disorders, family characteristics, social support, high relational mobility or very low tightness, bereavement, social isolation, etc.),
• Impact of mass media and social media on psychological attitudes and behaviors in the context of the COVID-19 emergency,
• Coping as a health professional during the epidemic (e.g., emotions, psychological burdens, anxiety, traumatic experiences, post-traumatic stress disorder),
• Clinical and health-based psychological interventions for sufferers, high-risk individuals, and those living in worst-hit communities,
• Clinical emergency protocols to manage mental health problems: evidence-based suggestions and recommendations to governments and policymakers,
• Behavior-change interventions to improve adherence to and compliance with preventive regulations and guidance,
• Internet interventions, remote psychological support, mHealth–eHealth-based treatments, and psychology-oriented digital tools and apps in the COVID-19 emergency,
• Monitoring changes in psychological, behavioral, and interpersonal responses to the COVID-19 emergency over time, and
• Cross-cultural comparisons in responding to and coping with the COVID-19 emergency at the individual, family, and interpersonal levels.

Rapid response by the psychological scientific community is necessary; thus, due to the exceptional nature of the COVID-19 situation, Frontiers is waiving all article publishing charges for COVID-19-related research.

## Data Availability Statement

The original contributions presented in the study are included in the article/supplementary material, further inquiries can be directed to the corresponding author/s.

## Author Contributions

All authors listed have made a substantial, direct and intellectual contribution to the work, and approved it for publication.

## Conflict of Interest

The authors declare that the research was conducted in the absence of any commercial or financial relationships that could be construed as a potential conflict of interest. The handling editor is currently organizing a Research Topic with the authors, and confirms the absence of any other collaboration.
